# Cultural Theories of Postpartum Bleeding in Matlab, Bangladesh: Implications for Community Health Intervention

**DOI:** 10.3329/jhpn.v27i3.3380

**Published:** 2009-06

**Authors:** Lynn M. Sibley, Daniel Hruschka, Nahid Kalim, Jasmin Khan, Moni Paul, Joyce K. Edmonds, Marjorie A. Koblinsky

**Affiliations:** ^1^ Center for Research on Maternal and Newborn Survival, Nell Hodgson Woodruff School of Nursing, Emory University, Atlanta, GA, USA,; ^2^ Santa Fe Institute, Santa Fe, New Mexico, USA,; ^3^ Reproductive Health Unit, Public Health Sciences Division, ICDDR,B, GPO Box 128, Dhaka 1000, Bangladesh

**Keywords:** Anthropometry, Cultural, Maternal mortality, Morbidity, Postpartum haemorrhage, Bangladesh

## Abstract

Early recognition can reduce maternal disability and deaths due to postpartum haemorrhage. This study identified cultural theories of postpartum bleeding that may lead to inappropriate recognition and delayed care-seeking. Qualitative and quantitative data obtained through structured interviews with 149 participants living in Matlab, Bangladesh, including women aged 18-49 years, women aged 50+ years, traditional birth attendants (TBAs), and skilled birth attendants (SBAs), were subjected to cultural domain. General consensus existed among the TBAs and lay women regarding signs, causes, and treatments of postpartum bleeding (eigenvalue ratio 5.9, mean competence 0.59, and standard deviation 0.15). Excessive bleeding appeared to be distinguished by flow characteristics, not colour or quantity. Yet, the TBAs and lay women differed significantly from the SBAs in beliefs about normalcy of blood loss, causal role of the retained placenta and malevolent spirits, and care practices critical to survival. Cultural domain analysis captures variation in theories with specificity and representativeness necessary to inform community health intervention.

## INTRODUCTION

Haemorrhage is the leading cause of maternal deaths worldwide (1-2). Of the 14 million women who suffer severe postpartum haemorrhage (PPH) (>1,000 mL of blood loss) every year, 140,000 die, and 1.6 million survive with long-term disability due to anaemia (3). Response to PPH is time-sensitive. Yet, more than 60 million women give birth in the home, mostly in developing countries and in underserved areas, attended by family members, neighbours, or traditional birth attendants (TBAs) (4). These care givers are usually ill-equipped to identify and manage PPH, and rates of referral to more expert care are often low, even where referral is possible (5). Adverse outcomes due to PPH are almost exclusively in low-resource settings where birth in the home is common and emergency care is scarce. This presents a daunting challenge for global safe motherhood. Targeting PPH in these vulnerable populations with effective, culturally-appropriate interventions is necessary to achieve Millennium Development Goal 5, which aims at reducing maternal mortality by 75% by 2015 (6-9).

The Delay Model has guided safe motherhood programming and research for over a decade (10), and it provides a useful framework for exploring the relevant context for life-threatening complications, such as PPH (10). This model proposes that delays in obtaining appropriate emergency care occur at three points: decision to seek care (predicated by recognition of problem), reaching care, and receiving emergency obstetric care. Each delay is influenced by a combination of sociocultural, economic, environmental and health service factors. The premise is that a reduction in the delays will lead to decreased mortality and morbidity.

There is a paucity of research focusing on the first delay—decision to seek care in the event of PPH (8-9). To address this gap, during 2005-2006, the Center for Research on Maternal and Newborn Survival of Emory University and the International Centre for Diarrhoeal Disease Research, Bangladesh (ICDDR,B) initiated a study among childbearing women and maternity-care providers in Matlab, Bangladesh. The overall aim of the study was to improve our understanding of recognition of and response to PPH and to use this understanding for informing health communications, provider-training, and future research.

Important to recognition of and response to PPH, and the focus of this paper, is the system of beliefs or cultural theories that women and home-based care givers have about the signs, causes, and treatment of normal and abnormal postpartum bleeding. Using cultural domain analysis, we describe here these cultural theories and identify clinically-important differences in knowledge about signs, causes, and treatments for PPH among professional skilled birth attendants (SBAs), TBAs, and community lay women.

## MATERIALS AND METHODS

### Location of study

In Bangladesh, approximately 90% of women deliver in the home, and 86% deliver with unskilled attendants. PPH remains a major cause of death (11). ICDDR,B supports research through its Matlab health services research area, located 56 km southeast of Dhaka. Since 1966, ICDDR,B has maintained a demographic and health surveillance system (HDSS) that covers an area dominated by a relatively-poor, rural population of about 220,000 whose main economic activities are farming and fishing. A community hospital at Matlab township, with four subcentres, provides 24-hour care, covering half of the population. In 2005, about 2,700 births took place in this area, and of these births, 52% occurred in the home (12).

### Procedures

#### Preliminary phase

The study had two phases—preliminary and main. In the preliminary phase, locally-salient terms for signs, causes, and treatments of normal and abnormal postpartum bleeding were derived from semi-structured successive free-listing interviews within a stratified random sample of women, aged 18-49 years, who delivered in the previous year (2005), women aged 50+ years living in an extended family and likely to be influential in matters of childbirth, TBAs, and SBAs (n=80, 20 per subgroup). In the Matlab context, the SBAs included professional and paraprofessional health workers (doctors, nurses, midwives, lady health visitors, and family welfare visitors). The sampling frames for the non-healthcare providers were lists of local residents contained in the HDSS of ICDDR,B. The sampling frames for TBAs and SBAs were lists of local traditional and professional care providers. The intent of the sampling strategy was to capture the maximal breadth and depth of terms. All terms mentioned by at least 20% of non-SBA informants and biomedical terms consistently mentioned by the SBAs or of theoretical interest, were retained and used for constructing the structured interview instrument for the main phase of the study. Further details of this preliminary phase of the study were reported elsewhere (13).

### Main phase

The retained terms were systematically linked via semantic relationships using a sentence frame substitution format (‘x' is a sign of/cause of/treatment for ‘y') and incorporated into a structured interview instrument consisting of 234 yes/no questions, arranged in a random order and focusing on three bleeding conditions—normal, abnormal excessive, and scanty (14). The instrument also included six open-ended questions about quantity of life-threatening bleeding at birth and within 24 hours of birth, and questions about personal experience with PPH and sociodemographic characteristics.

The structured interview was administered to a mixed sample comprising 19 women aged 15-49 years,19 women aged 50+ years, 20 TBAs, and 14 SBAs who participated in the free-listing interviews (described above). To test hypotheses about the social transmission of beliefs, the subject of another paper (15), we also purposively identified, screened, and interviewed 20 women who experienced excessive bleeding within 24 hours of birth in the home during the previous year (henceforth called ‘focal females'). We also interviewed up to three individuals in the women's social network perceived as influential in their childbirth, including 20 female relatives, 20 female neighbours, and 17 TBAs. The total sample of 149 informants, thus, combined eight subgroups of roughly equal proportions. Focal females were identified through the HDSS and complementary community health information system and screened using a PPH-specific diagnostic algorithm developed by the World Health Organization (WHO) for verbal autopsy (16). Focal females self-identified their social network members.

Four female social scientists conducted interviews in the local language. Coded responses were verified against audiotape recordings. Demographic and social data were entered into the computer using the SPSS software (version 15.0) (SPSS Inc., Chicago, IL, USA) and described using simple statistics. Inter-informant agreement data were entered into the computer using the UCINET software (version 6.0) (Analytic Technologies, Boston, MA, USA) for analysis.

### Statistical analysis

The statistical analysis involved three steps. First, to identify if there is a population-wide consensus in cultural theories about PPH held by the community TBAs and lay women, we excluded SBAs and fit a cultural consensus model (17) to the remaining sample of 135 informants. A consensus model is based on factor analysis and has three assumptions: (a) within a given domain of knowledge, there is a single, culturally-‘correct' way to respond (common truth), (b) individuals respond independently of each other (local independence), and (c) ability of each respondent to answer correctly is constant over all questions (homogeneity of items). Variation in response was modelled as the differential ability of the informants to give the culturally-correct response (individual competence). Two outputs from the factor analysis of the matrix of informant-by-informant agreement (on 234 questions, response-matching corrected for 50% guessing) were used for providing a check on whether the model assumptions are met: the first-to-second eigenvalue ratio should be >3:1, indicating that a single factor is more important than other factors in accounting for systematic variation in the agreement matrix, and individual loadings on the first factor should be positive, indicating general agreement with this single factor.

Second, to identify particular questions on which there is a high agreement—both positive and negative, we examined the patterns of responses to the set of 234 questions. For the sake of simplicity, we defined agreement as the probability of a true ‘yes' response either greater than 2/3 (agree ‘yes') or less than 1/3 (agree ‘no') and used Bayes' theorem to calculate the probability of agreement or disagreement on each question given the observed responses (18). We chose a probability of agreement greater than 90% as a cut-off for deciding when there was an agreement on a particular question.

The cut-off values were 0.72 (agree ‘yes') and 0.28 (agree ‘no') for the 135 informants. The choice of cut-off was somewhat arbitrary. An investigator could derive a different cut-off depending on the acceptable number of false positives, and the degree of consistency deemed necessary for ‘agreement' based on the aims of the study.

Third, we explored variation within the sample to identify the statistically significant and clinically important differences in knowledge content among the subgroups. Since we were unable to detect significant differences in responses within either the two TBA subgroups or within the five subgroups of non-healthcare providers, we combined these into a single TBA subgroup (n=37) and a single lay women subgroup (n=98) to simplify the analysis.

• To determine if agreement is greater among individuals within subgroups (SBAs, TBAs, lay women) on the 234 questions, we conducted a linear regression to predict the agreement between pairs of individuals, based on membership of subgroup. Since the observations included in the analysis were not independent, we used a quadratic assignment procedure for accounting for non-independence and for obtaining appropriate standard errors and p values (19-20). In the regression, the independent variables for category of caregiver included a set of three dummy variables: whether both individuals were SBAs, both TBAs, and both lay women. The dependent variable was proportion of agreement between any two individuals. We controlled for age, parity, and education of the informant and interviewers in the analysis.
• To determine if there are significant differences between subgroups (SBAs, TBAs, lay women), we compared the proportion of ‘yes' responses using a Bonferonni-corrected (21) Fisher's exact test (adjusted alpha=0.05/234 questions/3 subgroups=0.0000712).
• To identify particular questions on which there is a high agreement within subgroups, we repeated the procedure described previously. However, we recalculated the cut-off values to account for subgroup sample size. The cut-off values for SBAs were 0.93 and 0.07, for TBAs 0.78 and 0.22, and for lay women 0.73 and 0.27.

### Protection of human subjects

The study was approved by the IRB of Emory University and Ethical Review Committee of ICDDR,B. Voluntary verbal informed consent was obtained following the standard disclosure procedures.

## RESULTS

### Characteristics of informants

Most informants self-identified as Muslim while a small minority was Hindu (Table 1). On average, the TBAs were more than 10 years older, were less likely to have progressed beyond primary school, and had substantially lower incomes. The SBAs had the lowest parity and the highest educational level and income. Among the lay women, women of reproductive age were similar in parity and educational level as were elder influential women and relatives.

**Table 1.** Characteristics of respondents

Characteristics	SBA (n=14)	TBA (n=37)	All-lay women (n=98)	Subgroups of lay women				
				WRA (n=19)	EIW (n=19)	FF (n=20)	REL (n=20)	NBR (n=20)
Age (years)	42.0	52.6	41.5	28.4	63.3	27.4	50.4	39.1
Mean (SD)	(8.0)	(11.5)	(16.2)∗	(6.5)	(4.9)	(6.4)	(13.7)	(9.8)
Parity	2.1	5.7	4.4	2.6	6.8	2.8	10.2	4.3
Mean (SD)	(0.8)	(2.4)	(2.4)∗∗	(1.4)	(2.2)	(1.4)	(21.3)	(1.3)
Educational level								
No school	0.0	59.5	40.6	15.8	84.2	10.0	65.0	30.0
1-5 year(s)	0.0	37.8	30.2	36.8	15.8	40.0	15.0	45.0
6-10 years	35.7	2.7	24.0	42.1	0.0	40.0	15.0	20.0
11-12 years	35.7	0.0	4.2	0.0	0.0	0.0	0.0	0.0
Graduate school	28.6	0.0	1.0	5.3	0.0	10.0	5.0	5.0
Income (Tk) (%)								
<2,500	0.0	43.2	32.3	36.8	42.1	20.0	40.0	20.0
2,500-5,000 >5,000	0.0	46.0	33.3	47.4	36.8	35.0	20.0	35.0
>5,000	100.0	2.7	34.4	15.8	21.1	45.0	40.0	45.0
Religion (%)								
Hindu	7.1	10.8	12.5∗	10.5	21.1	10.0	10.0	10.0
Muslim	92.9	89.2	87.5	89.5	78.9	90.0	90.0	90.0

∗2 missing

∗∗3 missing

EIW=Elder influential women; FF=Focal females who gave birth in 2005 and experienced postpartum haemorrhage; NBR=Focal female friend or neighbour; REL=Focal female relative; SBA=Skilled birth attendant; SD=Standard deviation; TBA=Traditional birth attendant; WRA=Women of reproductive age, who gave birth in 2005

### Fit of cultural consensus model

The factor analysis indicated that a single factor was more important than other factors in accounting for systematic variation in the matrix of informant-by-informant agreement (eigenvalue ratio, 5.5). Moreover, there was a general agreement with this single factor (factor loadings, all positive). These results suggest a generally-shared cultural model of normal and abnormal postpartum bleeding. The average level of agreement among all pairs of informants was 59% (mean competence 0.59, standard deviation [SD] 0.15).

### Cultural theories of PPH

Table 2 shows the proportion of ‘yes' responses on questions about signs of normal and abnormal bleeding, excessive, and scanty. Values equal to or greater that 0.72, shown in dark grey, indicate agree ‘yes'. Values equal to or less than 0.28, shown in light grey, indicate agree ‘no'. One can see that the informants characterized normal bleeding as bleeding that starts and stops (intermittent) and slow-flowing. Excessive bleeding was characterized as bleeding that is continuous, forceful, and associated with poor appetite, pallor, weakness, and fainting. Scanty bleeding was not well-characterized, except informants agreed that it is neither forceful, continuous, and clotted, nor fresh red in colour.

**Table 2.** Proportion of ‘yes' responses on questions about signs of normal and abnormal postpartum bleeding

Bleeding/related signs	Normal	Abnormal	
		Excessive (life-threatening)	Scanty
Bleeding signs			
Starts and stops	0.87	0.18	0.55
Slow-flowing	0.94	0.09	0.71
Forceful	0.07	0.95	0.07
Continuous	0.12	0.90	0.12
Clotted	0.36	0.66	0.19
Fresh, red	0.53	0.59	0.19
Dark, ashy	0.50	0.46	0.48
Related signs			
Poor appetite	0.13	0.88	0.24
Pallor	0.13	0.99	0.20
Weakness	0.13	1.00	0.22
Faintness (dizziness)	0.15	0.99	0.15
Falling unconscious	0.16	0.67	0.12
Blood clotted in womb	0.44	0.40	0.52

Values ≥0.72=Agree ‘yes' (dark grey) and ≤0.28=Agree ‘no' (light grey); n=135

Also included in the structured interview were questions about whether each bleeding condition and sign requires an urgent response. The numbers in parentheses, below, represent the proportion of the informants who agreed ‘yes' and who agreed ‘no'. Nearly all agreed that excessive (0.97), forceful (0.86), continuous bleeding (0.84), and bleeding with clots (0.78) require an urgent response while normal bleeding (0.00), slow bleeding (0.10), and bleeding that starts and stops (0.17) do not.

Amount of bleeding was measured by both volume and number of different local collection-devices soaked with blood (Table 3). The quantities considered life-threatening and expressed here in local measures (mean and SD) varied widely, for example 2.65+2.00 *sheer* (one *sheer* is roughly equivalent to one litre) at birth.

**Table 3.** Quantifying excessive bleeding

Measure	At birth		Within 24 hours of birth	
	Mean	SD	Mean	SD
*Sheer*(∼litre)	2.65	2.00	3.56	2.74
Jute-bag	3.11	1.86	-	-
Mat	3.41	2.54	-	-
*Nekra* (cloth pad)	-	-	8.32	6.92
Sanitary napkin	-	-	7.41	5.66

SD=Standard deviation

The informants differentiated causes of and treatments for different kinds of bleeding that require an urgent response (Table 4). They agreed that *alga batas* (malevolent spirits) is a cause of excessive, forceful and continuous bleeding and bleeding with clots. There was no clear pattern of agreement about whether atonic uterus (failure of the uterus to contract after birth), retained placenta, or a bad tear in the birth-area were causes, except that the retained placenta might cause clotted blood. The informants, however, believed that saline and/or injections and medicines, i.e. allopathic remedies, are treatments.

**Table 4.** Proportion of ‘yes' responses on questions relating to cause of and treatment for kinds of bleeding requiring an urgent response

Cause/treatment	Kinds of bleeding requiring urgent response			
	Excessive	Forceful	Continuous	Clotted
Cause				
*Alga batas* (evil spirits)	0.82	0.76	0.85	0.77
Atonic uterus∗	0.64	0.62	0.62	0.51
Retained placenta	0.41	0.36	0.38	0.22
Bad tear	0.40	0.40	0.40	0.33
Treatment				
Special foods	0.25	0.28	0.26	0.30
Amulet, blessing	0.32	0.20	0.33	0.30
Hot compress	0.30	0.27	0.27	0.45
Injection	0.87	0.75	0.79	0.64
Medicine (tablet)	0.95	0.88	0.86	0.81
Saline	0.90	0.84	0.86	0.60

Values ≥0.72=Agree ‘yes' (dark grey) and ≤0.28=Agree ‘no' (light grey)

∗Mouth of the womb does not close; n=135

There was a consensus among the informants that, if the bleeding problem was caused by *alga batas*, the family should seek help of a traditional healer (*kobiraj*), and the problem should be treated with spiritual means, i.e. blessings or amulets (Table 5). On the other hand, if the bleeding problem was caused by atonic uterus, retained placenta, or a bad tear, the family should seek care of a trained allopathic provider (‘big' doctor), and the condition should be treated with medicines, injections and/or intravenous saline. Additional allopathic treatments included stitching for a tear. There was a little consensus about the role of the untrained allopathic provider (village doctor) in the management of these conditions.

**Table 5.** Proportion of ‘yes' responses on questions relating to treatment and healthcare-seeking for different causes of postpartum bleeding

Treatment/healthcare-seeking	Causes of bleeding			
	*Alga batas*	Atonic uterus	Retained placenta	Bad tear
Treatment				
Special foods	0.19	0.25	0.14	0.15
Amulet, blessing	0.91	0.42	0.14	0.07
Hot compress	0.36	0.54	0.21	0.63
Injection	0.24	0.76	0.79	0.64
Medicine (tablet)	0.36	0.82	0.73	0.81
Saline	0.29	0.57	0.87	0.31
Stitching	--	--	--	0.95
Manual removal	--	0.27	0.71	--
Forced gagging	0.10	0.07	0.70	0.03
Breastfeeding	0.07	0.28	0.23	0.03
Abdominal massage	0.44	0.33	0.51	0.07
Healthcare-seeking				
‘Big' doctor	0.27	0.87	0.93	0.96
Village doctor	0.41	0.64	0.54	0.45
*Kobiraj*	0.89	0.20	0.13	0.07

Values ≥0.72=Agree ‘yes' (dark grey) and ≤0.28=Agree ‘no' (light grey); n=135

### Variation in cultural theories of PPH

**Fig. 1.**
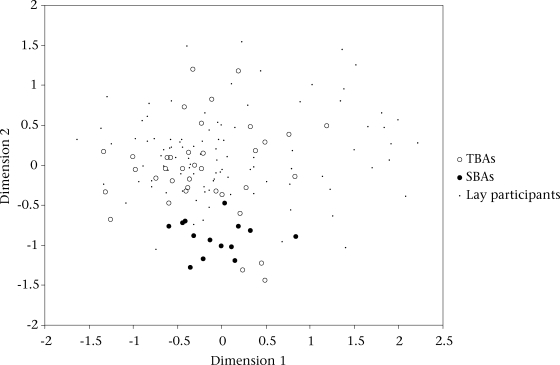
MDS scaling of participant agreement on 234 questions by role similarity (stress=0.21)

Examining within the agreement between the subgroups, we found that there was a significant increase in agreement among the SBAs (mean 0.78, SD 0.05, p<0.001) and to a lesser extent among the TBAs (mean 0.69, SD 0.06, p<0.01) over the level of agreement observed among unrelated pairs of individuals (mean 0.67, SD 0.07). The best two-dimensional scaling plot of inter-informant agreement illustrated distinct, yet overlapping, patterns of the SBA and TBA responses. The lay women appeared more closely aligned with the pattern of TBAs (Fig. 1).

MDS=Multidimensional scaling; SBAs=Skilled birth attendants; TBAs=Traditional birth attendants

Examining the agreement between the subgroups, we found that the SBAs differed significantly from the TBAs on 6% of the questions (15/234 questions) and from the lay women on 9% of the questions (20/234 questions, including 13 of the former). Notably, the TBAs and lay women did not differ significantly on any question. The observed differences mostly concerned the causal role of *alga batas* and retained placenta in excessive bleeding and the kind of care given if such bleeding was thought to be caused by either *alga batas*, retained placenta, or atonic uterus (Table 6).

**Table 6.** Questions where SBA's responses differ from TBA's and lay women's

Question text	Agreement		
	SBAs	TBAs	Lay women
*Alga batas*-related			
Is *alga batas* a cause of excessive, life-threatening bleeding?∗	0.00	0.78	0.84
Is *alga batas* a cause of bleeding with clots?∗	0.07	0.73	0.80
Is *alga batas* a cause of forceful bleeding?∗	0.07	0.78	0.76
Is *alga batas* a cause of continuous bleeding?∗	0.00	0.78	0.88
Is *alga batas* a cause of dark ashy bleeding?∗	0.00	0.86	0.85
Are bleeding problems caused by *alga batas* treated with amulets or blessings?	0.29	0.78	0.96
When a bleeding problem is caused by *alga batas*, does the family seek the help of a ‘big' doctor (at the health clinic)?	0.86	0.38	0.23
When a bleeding problem is caused by *alga batas*, does the family seek the help of a*kobiraj*?	0.29	0.73	0.96
Is a bleeding problem caused by *alga batas* treated by having a woman breastfeed her baby immediately after delivery?	0.50	0.08	0.07
Atonic uterus-related			
Is mouth of womb not closing (atonic uterus) treated by having a woman breastfeed immediately after delivery?∗	0.93	0.32	0.26
Is mouth of womb not closing (atonic uterus) treated by firmly massaging a woman's lower abdomen?∗	0.93	0.24	0.37
Retained placenta-related			
Is retained placenta a cause of forceful bleeding?∗	0.93	0.32	0.37
Is retained placenta a cause of bleeding with clots?∗	0.93	0.16	0.24
Is retained placenta a cause of excessive, life-threatening bleeding?∗	1.00	0.30	0.45
Is retained placenta a cause of continuous bleeding?†	0.93	0.30	0.41
Is retained placenta a cause of too little bleeding?†	0.00	0.62	0.51
Is retained placenta treated by having a woman breastfeed immediately after delivery?∗	1.00	0.30	0.21
Other			
Is bleeding to cleanse the womb of old blood after birth necessary for the woman to be healthy?∗	0.29	0.95	0.92
Do women who have a lot of children have a greater chance to have excessive, life-threatening bleeding at the time of birth?	1.00	0.32	0.14

∗Significant difference between both SBAs and TBAs and between SBAs and lay women

†Significant difference between SBAs and TBAs only. All others significant between SBAs and lay women only. Agreement among 14 SBAs for valuesw≡ or ≥0.93 and ≡ or ≤0.07; among 37 TBAs for values ≥ 0.78 and ≤ 0.22 and among 98 lay women for values ≥ 0.73 and ≤0.27, where agree ‘yes' (dark grey) and agree ‘no' (light gray)

SBA=Skilled birth attendant; TBA=Traditional birth attendant

In Table 6, the questions marked with an asterisk (∗) indicate a significant difference in responses between the SBAs and the TBAs and also between the SBAs and the lay women, and the questions marked with a cross (†) indicated a significant difference between the SBAs and the TBAs only. The questions without a mark indicate a significant difference between the SBAs and the lay women only. Within the subgroups, the SBAs unanimously agreed that *alga batas* was not a cause of excessive, forceful or continuous bleeding; however, most TBAs and lay women believed that it was. If the bleeding problem was thought to be caused by *alga batas*, most lay women believed that they should go to a *ko biraj*, not a trained allopathic provider. Moreover, the TBAs and lay women agreed that the problem should be treated with amulets and special blessings.

There was a strong agreement among the SBAs that the retained placenta was a cause of excessive bleeding. In contrast, the TBAs and lay women showed no clear agreement on this. The SBAs unanimously agreed that breastfeeding immediately after birth is a treatment for this condition; there was no clear agreement among the TBAs, and most lay women did not believe this.

Additionally, some SBAs thought that bleeding to cleanse the womb of old blood after birth is necessary for a woman's health whereas most TBAs and lay women shared this belief. And, while the SBAs unanimously agreed that high parity was a risk factor for PPH, there was no clear agreement among the TBAs on this issue, and the lay women agreed that parity was not a risk factor.

## DISCUSSION

This study described a significant variation in clinically-relevant cultural theories of postpartum bleeding held by Bangladeshi SBAs, TBAs, and lay women. The data indicate a generally-shared model of postpartum bleeding among the TBAs and lay women, in which excessive bleeding appeared to be most uniformly characterized by type of blood-flow and related signs, not colour or quantity. While there was a strong agreement that excessive, forceful or continuous bleeding and bleeding with clots require an urgent response, the wide differences of opinion about what constitutes an excessive, life-threatening quantity of bleeding at birth and within 24 hours of birth suggest that most informants had difficulty in quantifying loss of blood. Moreover, the quantities reported as life-threatening greatly exceeded the clinical definition of PPH of equal to or greater than 500 mL. Finally, the data suggest that the informants were uncertain about the aetiology of bleeding requiring an urgent response, apart from *alga batas*. However, they agreed what to do in response to such bleeding and also what to do when the cause of bleeding is known.

From a clinical perspective, the beliefs held by the TBAs and lay women may be critical for maternal survival. Notable for their potential influence on recognition of and response to PPH are that: (a) bleeding to cleanse the womb of old blood is necessary for health, coupled with an inability to quantify bleeding, (b) limited awareness of atonic uterus and retained placenta as causes of excessive bleeding and potentially useful first-aid measures, and (c) attribution of excessive bleeding to *alga batas* and importance of traditional healers and spiritual remedies. These beliefs also have been reported in Bangladesh (22-24) and elsewhere (25-26).

Findings from detailed illness narratives of recognition of and response to PPH among the 18 focal females and their care givers corroborate and extend these findings. While profuse bleeding and/or continuous bleeding were alarming signs in eight of the 18 accounts, in a number of cases, a belief that postpartum bleeding is normal and necessary to cleanse the womb led to delays in recognition and care-seeking. One woman's mother and aunt stated:

If [daughter] did not [become unconscious], I would not have realized that a mother could die if she bleeds too much. When we saw that [niece] was soaked over by blood, and she also shivered and then fell unconscious, we became panicked. Then we recognized that it is bad [Case 10].

The retained placenta, reported in six cases, was considered dangerous in itself (unrelated to excessive bleeding). One woman's mother noted:

We felt fear by seeing the retained placenta. What will we do now? That was our only fear. Sometimes women die if the placenta goes upwards to the liver. And bleeding is usual, after delivery bleeding is usual [Case 14].

Moreover, the retained placenta was initially treated with home-remedies, which exacerbated or caused bleeding and delayed care-seeking. These remedies included tying a sash around the abdomen to prevent the placenta rising up, inducing gagging, pressing on the lower abdomen, shaking and pulling the umbilical cord, and attempted manual removal. As one woman who assisted in her sister-in-law's birth recalled:

I trembled and fought with my nerves and tried to [remove] the placenta…. I was trying not to be upset but the placenta was not coming. We called her [aunt/TBA] in. She also inserted her hand [into the vagina]. She was checking whether the placenta would come. But it did not come… and then the profuse bleeding started. Fresh blood poured out [Case 1].

On the other hand, *alga batas* was mentioned as a cause of excessive bleeding in only one case narrative, and spiritual treatment was sought from a local traditional healer (*kobiraj* or *hujur*) in only two of the case narratives. This finding raises questions about beliefs in malevolent spirits as real barriers to timely emergency care for PPH and suggests the importance of triangulating normative with behavioural data.

### Strengths and limitations

The analytic approach to cultural domain analysis used in this study is sensitive to specific cultural theories about the signs, causes, and treatments for normal and abnormal postpartum bleeding and employs the sampling procedures that permit us to characterize both local ‘expert' systems of beliefs in the population and how these beliefs might differ within and among subgroups of population. The approach offers advantages over rapid assessment techniques that use a suite of ethnographic methods that capture specific cultural theories but are not sensitive to variability in these theories (27-28). It also offers advantages over the assessment method of knowledge, practice and coverage surveys commonly used in child-survival programmes that, while sensitive to individual variability in health practices, is not designed to capture the system of beliefs underlying these practices (29). These advantages are important when there is a much variation in cultural knowledge in a given setting. In this study, sampling was facilitated by the established HDSS and complementary community health information system of ICDDR,B. While this well-developed research infrastructure is not found in many settings, there are techniques to rapidly sample communities with a degree of representativeness that could be integrated with the strategy described here (30). The approach takes more time than many rapid assessment techniques and may, thus, not be appropriate for evolving public-health emergencies, such as outbreaks of diseases, disasters, or refugee situations. Nevertheless, there is a wide range of public-health problems that would benefit from the more thorough assessment strategy. The approach is also appropriate for the evaluation of changes in cultural knowledge.

A limitation is that the approach focuses on informants' knowledge of signs, causes, and treatments of normal and abnormal bleeding. While this suggests problems that may arise in relation to recognition of and response to an actual PPH event, complementary case studies should be used for corroborating the importance of such beliefs. Finally, due to the small SBA sample size, we were only able to identify substantial differences between subgroups that involved comparisons with SBAs. This limitation is offset by the fact that, in this study, we were primarily interested in substantial differences among subgroups. However, given our definition and parameters of agreement, we were only able to identify near-unanimous agreement among SBAs.

### Implications

The findings of this study have implications for health communications, provider-training, and future research.

In terms of health communications, TBAs and lay women are the obvious target groups in the Matlab setting. The data suggest content areas that could be emphasized, including that while some bleeding is normal after childbirth, it is important to watch for excessive bleeding, if forceful and/or continuous bleeding after birth is excessive; a retained placenta, or a womb that does not become firm after birth are leading causes of excessive bleeding; excessive bleeding after childbirth requires immediate skilled care, regardless of the cause, including *alga batas*; and finally, because any woman may experience excessive bleeding, timely recognition and response are lifesaving measures.

In terms of training, home-birth care givers need to be sufficiently skilled to prevent PPH through safe management of normal labour, especially the third stage of labour, and to monitor loss of blood at birth and in the vulnerable early postpartum period. Moreover, in the absence of uterotonic medications, care givers must be able to apply simple, potentially-effective measures for atonic uterus and to refer to a higher level of care if available and when these measures are not effective.

Given the difficulty in quantifying loss of blood, beyond flow characteristics, future research should include studies to validate simple, inexpensive diagnostic aids, such as special pad (Quaiyum MA. Personal communication, 2008), cloth (31), or drape (32) and to assess the introduction and impact of their use on practices of caregivers. Also needed are studies to assess the most effective strategies to teach home-based lifesaving skills, and their effect on postpartum blood loss (6-7).

On the basis of the findings of the cultural domain analysis and the focal female case narratives (data not presented in this paper apart from the examples), we have included new key messages and modified the pictorial materials contained in the intervention on home-based lifesaving skills (6). It is a component of the ICDDR,B/Matlab comprehensive maternal, neonatal and child healthcare programme, placing an increased emphasis on safe management of the third stage of labour and response to the retained placenta. The intervention on home-based lifesaving skills intentionally targets pregnant woman, her family care giver(s), and birth attendant, i.e. those who will be present during birth in the home and who must quickly recognize and respond to a life-threatening complication of the mother or the newborn.

In conclusion, the approach to cultural domain analysis used in this study permits a level of local specificity and representativeness that, we believe, are necessary to adequately inform the development of community health interventions. The approach can be corroborated through behavioural studies.

More generally, research on the role of beliefs in shaping perceptions of risk, problems, and treatments for PPH adds to the body of evidence of factors potentially influencing home-based care and a decision to seek care, such as cost, distance, and support systems. Returning to the Delay Model, a theoretical and practical issue raised in this study is the relative contribution of health-related beliefs, individual attributes, and socioecological constraints to care and care-seeking for obstetric emergencies. This represents a relatively-unexplored agendum in safe motherhood (8-9). Addressing this agendum is necessary if we are to improve programming to reduce maternal death and disability during the transition to skilled birth attendance for all women.

## ACKNOWLEDGEMENTS

This project was made possible through the Robert W. Woodruff Health Sciences Center Foundation, Emory University, Atlanta, Georgia, USA. The authors thank Lauren Blum and Roslyn Botlero for their assistance during the pre-testing phase of the study and Aasma Afroz, Nargis Farhana, and Shahana Parveen for their role in its implementation. The authors also thank Gery Ryan, Clarence Gravlee, and Joe Henrich for their advice in applying cultural consensus modelling to the problem of PPH. The authors are solely responsible for the views expressed in the paper, which, in no way, reflect the official opinion of the funding body.
